# Assessing the Importance of Variation in Diagnostic Coding Among the Three Countries in the UK Biobank

**DOI:** 10.1002/lrh2.70083

**Published:** 2026-05-05

**Authors:** Lei Clifton, Wenyu Liu, Jennifer A. Collister, Thomas J. Littlejohns, Raphael R. Goldacre, Naomi Allen, David J. Hunter

**Affiliations:** ^1^ Nuffield Department of Primary Care Health Sciences University of Oxford Oxford UK; ^2^ Nuffield Department of Population Health University of Oxford Oxford UK; ^3^ UK Biobank Ltd UK; ^4^ Department of Epidemiology Harvard TH Chan School of Public Health Boston Massachusetts USA

**Keywords:** country variation, dementia, diagnostic coding, Parkinson's disease (PD), type 2 diabetes (T2D), UK Biobank (UKB)

## Abstract

**Background:**

The UK Biobank (UKB) study has linked hospital inpatient data collected from England, Scotland, and Wales, which use different clinical coding systems to record health outcomes. Scotland records up to 6 different diagnostic codes for one inpatient episode, compared with up to 20 in England and 14 in Wales. We assessed the relationship of the variations in diagnostic coding among countries on observed disease incidence rates.

**Methods:**

We examined the number of diagnoses coded by each country, and then compared the incidence of three diseases between countries: Parkinson's disease (PD), type 2 diabetes (T2D), and dementia. We constructed Cox models for each disease, adjusting for “country.”

**Results:**

Compared with England, Scotland appears to have the lowest risk (hazard ratio, HR) for all three diseases: HR [95% CI] = 0.62 [0.54, 0.72] for PD, 0.49 [0.45, 0.54] for T2D, and 0.88 [0.78, 0.99] for dementia.

**Conclusions:**

The observed incidence of these diseases and the estimated effect of “country” in Cox models are likely influenced by the clinical coding variations among countries. Researchers need to be aware of this and account for these variations in their analyses.

## 1 Introduction

Routinely collected health statistics in the United Kingdom play an important role in population health research, health resource allocation, and descriptive and observational epidemiology. A major data source is hospital inpatient data that contains information about reasons for hospitalization and additional diagnoses not necessarily related to the hospitalization. These hospital inpatient data are recorded in the Hospital Episode Statistics (HES) for England, Scottish Morbidity Record (SMR), and Patient Episode Database for Wales (PEDW). We will collectively refer to all these three sources of hospital inpatient data as Admitted Patient Care (APC). The disease diagnostic (e.g., ICD‐10) codes in APC are currently the main source of health outcome ascertainment for researchers. In England and Wales, an hospital **“**episode” is a continuous period of care for a patient under a single consultant within one hospital. If the patient is transferred to a different consultant, a new episode begins. One or more of these episodes make up an hospital **“**spell,” which is the patient's entire stay in that hospital from admission to discharge. The inpatient data from Scotland also contains “episodes,” but they are not arranged into “spells” in this way. In all three countries, admitted episodes of patient care are assigned one main (primary) code for the reason for admission, as well as a variable number of secondary (or underlying) disease codes. The primary diagnosis in APC is recorded at “location 0”, with secondary diagnoses recorded at “location 1, 2, 3,…” However, Scotland records a maximum of 6 primary plus secondary diagnostic codes for one episode, compared with up to 20 in England, and 14 in Wales. In England the number of diagnosis fields has increased over time, from up to 7 codes before April 2002, up to 14 in April 2002 to March 2007, and up to 20 since April 2007 [1].

This variability in the coding practices for the APC data by country may lead to variation in the likelihood of conditions being recorded that are not the main (primary) reason for an admitted episode and yield mistaken inferences about the relative burden of diseases across countries. We sought to understand the likely association for three diseases that are more likely to be recorded as a secondary diagnosis: Parkinson's disease (PD), type 2 diabetes (T2D), and all‐cause dementia.

The UK Biobank (UKB) is an ongoing prospective cohort study of approximately 500 000 participants recruited from 22 assessment centres across England, Scotland, and Wales between 2006 and 2010 [2]. As primary care records are currently only available for approximately half of the cohort participants for a limited period of follow‐up, the APC records are used in most analyses to identify incident disease. The linkage to electronic hospital records in UKB offered the opportunity to evaluate whether differences in coding practices in the APC data between the three countries influence conclusions about regional variation in disease incidence. To the best of our knowledge, there have been no studies examining the importance of this variability.

## 2 Methods

We first examined the distribution of the number of “primary + secondary” ICD‐10 diagnostic records per episode in the APC records from the three countries, excluding duplicates within each episode and truncated at the earliest hospital inpatient data censoring date across all data providers (May 31, 2022).

For each individual, we defined “the first disease location” as the first occurred location of the disease diagnostic codes among all episodes of a patient. Within each episode, we find the first disease‐specific location among all the disease diagnoses, since a specific disease may be defined by several ICD codes. We then take the minimum location of these first locations among all episodes, since a patient may have multiple hospital stays which may contain multiple episodes. This method yielded, for each patient, a number for the “first disease location” for each disease.

For example, if dementia is the primary diagnosis (i.e., the main reason for admission) in any episode of an individual, their “first disease location” for dementia will be “location 0”. If a disease dementia is first recorded as a secondary diagnosis in an episode and then became a primary diagnosis (at location 0) in a later episode, the “first disease location” for dementia of that person will still be “location 0”. If the disease was never recorded as the primary diagnosis, the “first disease location” can be at “location 1, 2, 3,…,” whichever is the first occurred location across all episodes for that individual.

For PD and dementia, we used the outcome definition derived by the UKB (Resource 594). A T2D‐related diagnostic code list is currently not available from UKB. We instead used clinical codes as reported in the existing literature [3]. For description of analysis populations and outcome definitions, please see Supporting Information Section 3.

The variation in clinical coding between the three countries is specific to APC data. Therefore, we compared cases in APC data with the death records since the latter are not affected by between‐country differences in the number of available diagnosis code positions and are thus less susceptible to coding bias.

We then constructed Cox proportional hazards models to quantify and compare the effect of “country” alongside established risk factors on the risk prediction of each disease. We excluded prevalent cases at baseline, using APC and UKB self‐report data. For incident cases in our disease prediction Cox model, we used both APC and the Death Registry for accurate disease ascertainment, bearing in mind that cases recorded in different sources are not mutually exclusive. We included established disease‐specific risk factors available in UKB and an additional covariate “country” (reflecting the healthcare systems and the electronic medical record systems being used) in the Cox model for each disease [4].

## 3 Results

Of 502 358 participants, 444 908 have at least one hospital inpatient record, with 11%, 15%, and 12% participants in England (*N* = 445 718), Scotland (*N* = 35 836), and Wales (*N* = 20 804), respectively, not appearing in the APC data over the follow‐up period. Baseline characteristics by country are provided in Table S1.

**FIGURE 1:**
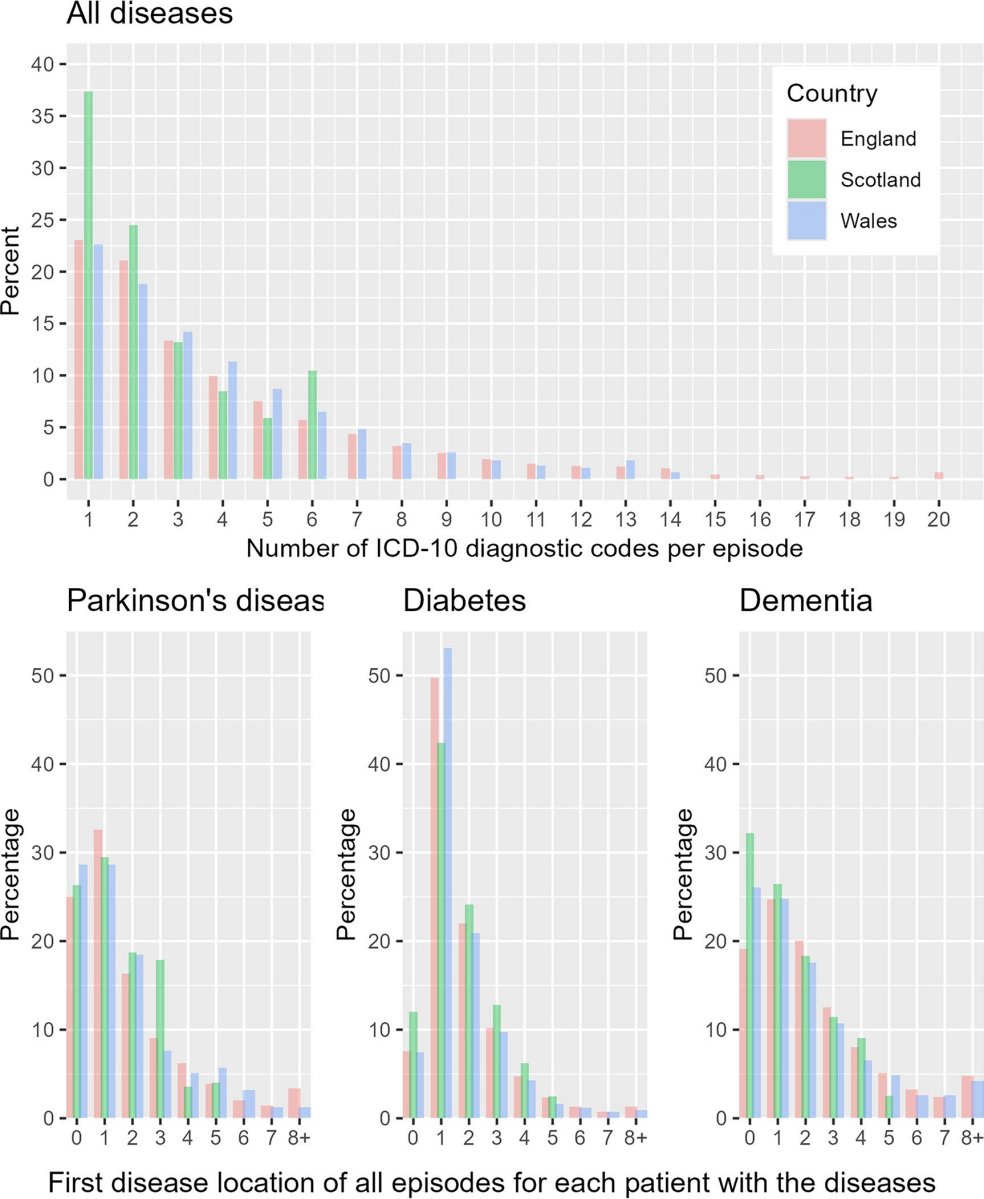
Top: Number of “primary + secondary” ICD‐10 diagnostic codes per episode, excluding duplicates within each episode and truncated at the earliest hospital inpatient data censoring date among countries. England records up to 20 diagnostic codes per episode, Scotland up to 6, and Wales up to 14. The sum of all counts is the number of episodes (not patients). For the raw number of ICD‐10 diagnostic codes per episode by country, please see Table S2. The median number of ICD‐10 diagnostic codes per episode is 3 for England and Wales, compared with 2 for Scotland. Detailed figures by age and sex are shown in Figure S1a–c. Bottom: First disease location of all available episodes for each patient with Parkinson's disease, diabetes, and dementia, respectively, by country. Scotland appears to have different recording patterns from England and Wales for PD and diabetes, but less so for dementia. We note that the disease location ranges from 0 to 19 for England (i.e., max 20 diagnostic codes per episode), from 0 to 5 for Scotland (i.e., max 6 diagnostic codes) and up to 13 for Wales (i.e., max 14 diagnostic codes), where primary diagnoses are recorded as “location 0” in the raw data. For brevity, the bottom figures show “8+” disease location.

Across all diseases recorded in APC, Scotland has a notably higher percentage (37%) of episodes with one diagnostic code (i.e., primary diagnosis only) than England and Wales (both approximately 23%, Figure 1 top). In England and Wales, 19.2% and 17.8% of diagnoses are recorded after the first six diagnostic codes, respectively. The median number of ICD diagnostic codes per episode is 3 for England and Wales, compared with 2 for Scotland. Figure S1a–c in the Supporting Information further shown the plots by age and sex. The corresponding number of ICD‐10 diagnostic codes per episode by country is tabulated in Table S2.

In England, Scotland, and Wales, respectively, the percentage of primary diagnoses is 25.0%, 26.3%, and 28.7% for PD; 7.6%, 12.0%, and 7.5% for T2D; and 19.1%, 32.2%, and 26.1% for dementia (Figure 1). T2D is less likely to be recorded as the primary diagnosis in all three countries, especially in England and Wales, compared with PD and dementia. Dementia is more likely to be recorded as the primary diagnosis in Scotland than in England and Wales, compared with PD and T2D. The pattern of the “first disease location” for PD is similar in all three countries.

The prevalent and incident cases in APC for each disease are shown in Table 1, with the incident cases identified through the Death Registries added for comparison. It shows that the APC disease percentages in Scotland are lower than England and Wales for PD and T2D, but not for dementia. Conversely, the death records indicate that the percentages for T2D and dementia are higher in Scotland than in England and Wales. Additionally, Table S12 shows the crude and age‐ and sex‐adjusted APC plus Death Registry incident rates (estimated using Poisson regression) per 100 000 person‐year by country for PD, T2D, and dementia. To take account of the different number of diagnostic codes in APC data among the three countries, we truncated the number of diagnostic codes in England and Wales to be six (the same as Scotland), and then compared the observed disease percentage between “all diagnostic codes” and “the first 6 codes only” in Tables S3–S5.

**TABLE 1:** Prevalent and incident cases recorded in APC and incident cases recorded in Death Registry for each disease by country.

		England (*N* = 445 718)	Scotland (*N* = 35 836)	Wales (*N* = 20 804)
Prevalent cases in APC	PD^a^	367 (0.1%)	23 (0.1%)	21 (0.1%)
	T2D^b^	10 091 (2.3%)	573 (1.6%)	499 (2.4%)
	Dementia^c^, ^d^	NA	NA	NA
Incident cases in APC	PD^a^	3953 (0.9%)	210 (0.6%)	137 (0.7%)
	T2D^b^	32 017 (7.2%)	1500 (4.2%)	1488 (7.2%)
	Dementia^c^, ^d^	8138 (1.8%)	650 (1.8%)	304 (1.5%)
Incident cases in Death Registry	PD^a^	1116 (0.3%)	106 (0.3%)	48 (0.2%)
	T2D^b^	1870 (0.4%)	317 (0.9%)	80 (0.4%)
	Dementia^c^, ^d^	2669 (0.6%)	385 (1.1%)	148 (0.7%)

*Note:* Each cell shows the number of cases (%), where percentage = number of cases/*N* in each country, where *N* is before applying disease‐specific exclusion criteria.

^a^ Parkinson's disease (PD) was based on the code lists for “algorithmically‐defined outcomes” (UKB Resource 460).

^b^ Type 2 Diabetes (T2D) was based on Eastwood et al. [3] Algorithm 1.

^c^ Dementia was based on the code lists for “algorithmically‐defined outcomes” (UKB Resource 460), where the self‐reported dementia was under the group of Dementia/Alzheimers/Cognitive Impairment.

^d^ For dementia, the number of patients aged 60 or above are: *N* = 194 490 in England; *N* = 14 658 in Scotland; and *N* = 8401 in Wales.

After applying the exclusion criteria (Figures S2–S4) to the 502 358 UKB participants, approximately 499 000, 473 000, and 204 000 participants are included in the study populations for PD, T2D, and dementia, respectively. The dementia population is restricted to individuals aged 60–70 years, as they are at greatest risk of developing dementia. Baseline characteristics for each disease after applying the exclusion criteria are shown in Tables S6–S8. Table 2 shows the estimated effect (hazard ratios, HR) of variable “country” obtained from Cox model after adjusting for known risk factors for each specific disease. For all three diseases, England (the reference category) has the highest risk, while Scotland has the lowest risk with HR = 0.62, 0.49, and 0.88, for PD, T2D, and dementia, respectively. These lower risks in Scotland are potentially misleading, as they are likely to be influenced by different healthcare systems and the coding variation across countries, especially for secondary conditions. The variable “country” is statistically significant (*p* < 0.0001) for PD and T2D, but not for dementia. One possible explanation is that compared with PD and T2D, dementia may be regarded as an important condition and therefore prioritized for coding; indeed, Scotland had the highest proportion of dementia as a primary diagnosis. The HR of established risk factors for the three disease outcomes was not materially affected when adjusted for “country” (Tables S9–S11). The proportional hazards assumption was met by visually assessing the scaled Schoenfeld residuals via the “survminer” R package.

**TABLE 2:** Hazard ratio (HR) of variable “country” obtained from Cox model after adjusting for known risk factors for each disease^a^.

	England	Scotland	Wales	*p*
PD	*N* = 442 577	*N* = 35 614	*N* = 20 739	
	1 (reference)	0.62 [0.54, 0.72]	0.77 [0.64, 0.92]	< 0.0001
T2D	*N* = 419 839	*N* = 34 014	*N* = 19 612	
	1 (reference)	0.49 [0.45, 0.54]	0.96 [0.89, 1.04]	< 0.0001
Dementia	*N* = 181 850	*N* = 13 773	*N* = 7983	
	1 (reference)	0.88 [0.78, 0.99]	0.98 [0.83, 1.16]	0.098

*Note:* HR is shown with its associated 95% confidence interval (CI). *N* is after applying disease‐specific exclusion criteria (shown in Figures S2–S4). Detailed results are in Tables S11–S13.

Abbreviations: PD, Parkinson's disease; *p* value, overall *p* value of variable “country”; T2D, type 2 diabetes.

^a^ Established risk factors included for PD: age, sex, self‐reported ethnicity, Townsend deprivation score quintile, family history of PD, and smoking. T2D: age, self‐reported ethnicity, Townsend deprivation score quintile, family history of diabetes, waist circumference (cm), BMI, physical inactivity, and hypertension. Dementia: age, sex, self‐reported ethnicity, Townsend deprivation score quintile, family history of dementia, smoking, APOE_e4_carrier, education, physical inactivity, depression, diabetes, hearing impairment, hypertension, social isolation, and BMI.

## 4 Discussion

The main strength of our study is the systematic examination of hospital episodes coded at admission and each time a patient moves between different hospital units using the large UKB cohort with approximately 15 years of follow‐up. One limitation is the relatively small numbers of participants in Wales and Scotland, compared with England. Nevertheless, the availability of in‐hospital patient data across the entire cohort has enabled us to examine the importance of disease coding variation among the three countries.

In general, Scotland displayed different recording patterns for inpatient diagnostic codes, compared with England and Wales. This is more prominent for PD and T2D, than dementia. In addition, Scotland has a higher percentage of people with no hospital records, and the majority of those who do have a hospital record only have one or two codes included. Variation in disease coding across jurisdictions in any study may influence the interpretation of research findings seeking to understand geographic differences in disease incidence. Here we demonstrate that differences in the maximum number of ICD codes routinely recorded in hospital inpatient records in England, Scotland, and Wales may be misinterpreted as differences in the incidence rates of a disease between these countries. For instance, a recent publication [5] using UKB data reported a lower risk of heart failure in urban Scotland than England. The authors speculated that this might be due to the Scottish population being densely concentrated in Glasgow and Edinburgh, thus potentially benefiting from easier access to health services. Our study provides another plausible explanation (i.e., coding variation across countries) for this phenomenon.

Similarly, a recent study [6] examining health disparities using UKB reported a higher relative burden of disease in England than in Scotland and Wales. The authors postulated that this may be attributed to higher socio‐economic deprivation (i.e., lower social economic status) for participants recruited from England compared to those from Scotland and Wales. However, we observed that 23.9% of UKB participants in Scotland were in the most deprived quintile (measured by the Townsend Deprivation index) compared with 19.9% of participants in England. Studies using data that is geographically determined such as air pollution exposure, or latitude, could be biased if geographical differences in disease ascertainment are not accounted for. There is no reason to suspect that the incidence of these diseases differs between countries. For example, the charity Parkinson's UK reports that the prevalence of disease is similar in England, Scotland and Wales (except for London which has, on average, the youngest population) [7]. Another report, produced as part of the Quest for Quality and Improved Performance (QQUIP) provided by The Health Foundation, also showed a similar prevalence of diabetes in the United Kingdom (3.9%, 3.7%, and 4.4% in England, Scotland, and Wales, respectively) [8]. An interactive map from Alzheimer's Research UK webpage shows similar estimated numbers of people aged 65 years and above who are living with dementia across the United Kingdom [9]. A 2‐year lower life expectancy in Scotland shown by Office for National Statistics suggests that, on average, the Scottish population is not healthier than that of England [10].

We examined this in the UKB, but the same issue would apply to any study examining incidence rates across these three countries. Although this issue is clearly flagged in the UKB documentation “Hospital inpatient data,” it could be easily overlooked. In the present analysis, we cannot exclude the possibility that differences in the health systems in the devolved nations in terms of how patients are managed at home or in a care home might also influence rates of hospitalization and thus recording of diagnoses. More specifically, while we have shown the differences in the percentage and where in the hospital record the diseases are coded, this does not explain the low recorded cases of PD/T2D in Scotland, which may, in part, be due to the different healthcare systems and electronic medical record practice that are unobservable or unmeasurable. The differences in the number of diseases coded alone may not fully explain the reduced risk of disease (e.g., HR = 0.6 for PD in Scotland compared with England shown in Table 2), but it can be served as a proxy for the unobservable variations among countries.

The increasing availability of large databases that can be used for both epidemiologic and health care policy studies has led to an explosion in publications in both domains. In addition to geographical variation in disease coding, we have previously noted [4] that conditions that are not often the principal cause of a hospitalization may have a lag of many years before a diagnosis recorded in the primary care record appears in the APC data, leading to an underestimation of incidences and age at onset of these conditions when solely using APC data for case ascertainment. Thus, researchers analyzing research resources like UKB need to understand the provenance of the data and rules that govern these databases in order to reach robust conclusions that are not influenced by artifacts and idiosyncrasies in the recording of the data.

## 5 Conclusion

Scotland appears to have the lowest risk (measured by HR) of PD, T2D, and dementia among the three countries. This is likely misleading, probably influenced by the coding variation (owing to fewer opportunities to record secondary diagnoses in hospital data from Scotland) and potentially other unobserved differences in clinical management between the three countries.

## Author Contributions

L.C. and D.J.H. conceived the project. L.C. and W.L. outlined the statistical methods. L.C., W.L., and D.J.H. drafted the manuscripts. W.L. conducted the statistical analysis. J.A.C. reviewed the R scripts by W.L. All authors have contributed to the study design, revised the manuscript, and agreed on its contents.

## Funding

This work was supported by Cancer Research UK (grant number C16077/A29186). The authors also acknowledge support from the Nuffield Department of Population Health, University of Oxford, UK. The UK Biobank study was supported by the Wellcome Trust, Medical Research Council, Department of Health, Scottish Government, and Northwest Regional Development Agency. It has also received funding from the Welsh Assembly Government and British Heart Foundation.

## Disclosure

The lead author affirms that this manuscript is an honest, accurate, and transparent account of the study being reported; that no important aspects of the study have been omitted; and that any discrepancies from the study as planned have been explained.

## Ethics Statement

The UK Biobank study received ethical approval from the North West Multi‐Centre Research Ethics Committee (REC reference: 11/NW/03820). The analyses presented here are based on existing data from the UK Biobank cohort study, and the authors were not involved in participant recruitment. No patients were asked to advise on interpretation or writing these results. Results from UK Biobank are routinely disseminated to study participants via the study website and social media outlets.

## Consent

All participants gave written informed consent before enrolment in the study, which was conducted in accordance with the principles of the Declaration of Helsinki.

## Conflicts of Interest

The authors declare no conflicts of interest.

## Supporting information


**Table S1:** Baseline characteristics of all UKB participants in England, Scotland, and Wales.
**Table S2:** Number of distinct primary + secondary diagnostic codes per episode by country, censored at the earliest UKB administrative censoring date among the three countries (May 31, 2022).
**Figure S1:** (a) Number of “primary + secondary” ICD‐10 diagnostic codes per episode (excluding duplicates) by sex and country for participants aged (40, 50). (b) Number of “primary + secondary” ICD‐10 diagnostic codes per episode (excluding duplicates) by sex and country for participants aged (50, 60), including 62 767 men and 81 278 women. (c) Number of “primary + secondary” ICD‐10 diagnostic codes per episode (excluding duplicates) by sex and country for participants aged (60, 70), including 95 134 men and 105 335 women.
**Table S3:** Parkinson's disease diagnostic cases and percentage by country, counting each disease once using the first disease location.
**Table S4:** Type 2 diabetes diagnostic cases and percentage by country, counting each disease once using first disease location.
**Table S5:** Dementia diagnostic cases and percentage by country, counting each disease once using first disease location.
**Figure S2:** Flowchart of study population for Parkinson's disease.
**Figure S3:** Flowchart of study population for type 2 diabetes.
**Figure S4:** Flowchart of study population for dementia.
**Table S6:** Population characteristics for Parkinson's disease.
**Table S7:** Population characteristics for type 2 diabetes.
**Table S8:** Population characteristics for dementia.
**Table S9:** Cox models for Parkinson's disease (*N* = 498 930).
**Table S10:** Cox models for type 2 diabetes (T2D) (*N* = 473 465).
**Table S11:** Cox models for dementia (*N* = 203 606).
**Table S12:** Crude and age‐ and sex‐ adjusted incident rates (estimated using Poisson regression) per 100 000 person‐year by country for PD, T2D, and Dementia.

## Data Availability

This research has been conducted using the UK Biobank Resource under Application Number 33952. Requests to access the data should be made via application directly to the UK Biobank, https://www.ukbiobank.ac.uk. The code used for analyses is available at https://gitlab.com/wenyul/countrydiff.
